# Dynamic evaluation of liver fibrosis to assess hepatocellular carcinoma risk in patients with chronic hepatitis B receiving nucleoside analogs treatment

**DOI:** 10.1590/S1678-9946202466027

**Published:** 2024-05-13

**Authors:** Jia Luo, Ming Yuan, Shan Li, Lijuan Chen, Mingsha Zhou, Hailan Li, Xiuyuan Bai, Zhiyu Zhang, Weiqi Zeng, Xueyi Sun, Qiongfang Zhang, Yi Chen, Li Zhou

**Affiliations:** 1 Chongqing Medical University, School of Public Health, Department of Epidemiology, Chongqing, China; 2 Chongqing Medical and Pharmaceutical College, Chongqing, China; 3 Chongqing Medical University, School of Public Health, Chongqing, China

**Keywords:** Chronic hepatitis B, Liver fibrosis, Noninvasive prediction model, Hepatocellular carcinoma, Dynamic evaluation

## Abstract

Despite good hepatitis B virus (HBV) inhibition by nucleoside analogs (NAs), cases of hepatocellular carcinoma (HCC) still occur. This study proposed a non-invasive predictive model to assess HCC risk in patients with chronic hepatitis B (CHB) receiving NAs treatment. Data were obtained from a hospital-based retrospective cohort registered on the Platform of Medical Data Science Academy of Chongqing Medical University, from 2013 to 2019. A total of 501 patients under NAs treatment had their FIB-4 index updated semiannually by recalculation based on laboratory values. Patients were divided into three groups based on FIB-4 index values: < 1.45, 1.45–3.25, and ≥ 3.25. Subsequently, HCC incidence was reassessed every six months using Kaplan-Meier curves based on the updated FIB-4 index. The median follow-up time of CHB patients after receiving NAs treatment was 2.5 years. HCC incidences with FIB-4 index < 1.45, 1.45–3.25, and ≥ 3.25 were 1.18%, 1.32%, and 9.09%, respectively. Dynamic assessment showed that the percentage of patients with FIB-4 index < 1.45 significantly increased semiannually (*P* < 0.001), and of patients with FIB-4 index ≥ 3.25 significantly decreased (*P* < 0.001). HCC incidence was the highest among patients with FIB-4 index ≥ 3.25. The FIB-4 index effectively predicted HCC incidence, and its dynamic assessment could be used for regular surveillance to implement early intervention and reduce HCC risk.

## INTRODUCTION

Hepatitis B virus (HBV) infection is a serious public health issue worldwide, affecting 257 million people^1^. Estimates indicate that about 887,000 people will die of chronic HBV infection-related diseases every year, including liver cirrhosis, hepatocellular carcinoma (HCC), and acute and chronic liver failure^2,3^. Chronic HBV infection is a major cause of cirrhosis and HCC^4^. Increasing evidence shows that recent advances in nucleoside analogs (NAs) therapy has achieved viral suppression, HBsAg reduction and improved treatment adherence in patients with chronic hepatitis B (CHB)^5-9^, thus reducing HCC development and liver-related death^10-12^. Despite such an excellent inhibitory effect, some HBV cases still develop HCC, thereby remaining a main public health issue in CHB-endemic areas. Hence, regular monitoring and identification of high-risk HCC development for early intervention is an important clinical issue even in patients treated with NAs.

Prior to the NAs, serum HBV DNA levels were an important risk factor for HCC development^13^. However, most patients treated with NAs can rapidly achieve a complete virologic response, thus the burden of liver fibrosis is a key factor in CHB patients’ progression to HCC^14^. Recent studies have shown that NAs treatment can induce regression or even reversal of liver fibrosis in patients with CHB, and the risk of HCC varies accordingly^15-18^. Longitudinal monitoring at different time points is therefore required to assess the liver fibrosis status of patients with CHB and consequently analyze the risk of HCC development.

Liver biopsy remains the golden standard for assessing liver fibrosis, but due to invasiveness, sampling error, and non-reproducibility limitations^19^, liver tissue biopsy does not allow regular longitudinal patient monitoring. Thus, selecting a simple and reliable noninvasive predictive model is essential for regular assessment of liver fibrosis stage. The FIB-4 index has been recommended by WHO and the Guidelines for the Prevention and Treatment of Chronic Hepatitis B^20^ for assessment and staging of liver fibrosis in patients with CHB within resource-limited settings. Moreover, the study by Tada *et al*.^21^ confirmed the validity of the FIB-4 index in predicting HCC incidence. Accordingly, the FIB-4 index was chosen to dynamically assess liver fibrosis in CHB patients.

This study evaluated liver fibrosis severity in CHB patients under NAs treatment based on the FIB-4 index, observed the dynamic changes of fibrotic burden in these patients , performed regular monitoring, and assessed HCC risk based on the FIB-4 index.

## MATERIALS AND METHODS

### Study population

Data was obtained from a hospital-based retrospective cohort study. A total of 1,539 patients were enrolled in the Platform of Medical Data Science Academy, Chongqing Medical University, from 2013 to 2019. Of the 1,038 patients excluded, 538 had not received NAs treatment or received treatment for less than 24 weeks, 355 had other liver diseases, and the remaining 145 patients had a history of HCC at baseline. Finally, 501 patients with CHB were included in the analysis. Inclusion and exclusion criteria were listed as follows:

Inclusion criteria:

1. Patients with CHB diagnosed based on the “Guidelines for the Prevention and Treatment of Chronic Hepatitis B” (2015, 2019);
2. HBsAg-positive for more than 24 weeks;
3. Patients who received NAs treatment and had good adherence.

Exclusion criteria:

1. Coinfection with hepatitis A virus, hepatitis C virus, hepatitis D virus, hepatitis E virus, or HIV;
2. Received NAs treatment for less than 24 weeks;
3. CHB combined with other autoimmune liver disease or metabolic liver disease;
4. Alcohol or drug abusers included in standardized follow-up;
5. Patients with a history of HCC.

### Baseline dates and outcomes

Date of the first CHB diagnosis during follow-up was used as the baseline date. Endpoint of follow-up was the last visit for HCC-free patients or the date of HCC diagnosis in 2019. This study outcome was the development of HCC during follow-up. HCC diagnosis was confirmed by histopathology or imaging criteria (computed tomography, magnetic resonance imaging or contrast-enhanced ultrasonography) according to the AASLD and EASL guidelines^22,23^.

During follow-up, patients’ laboratory tests and ultrasonography information were collected every 3–6 months. If the ultrasound detected nodular lesions, additional imaging tests (computed tomography, magnetic resonance imaging or contrast-enhanced ultrasonography) were performed. Indicators related to liver function included: platelet count (PLT), alanine aminotransferase (ALT), aspartate aminotransferase (AST), gamma-glutamyl transferase (GGT), albumin (ALB), alkaline phosphatase (ALP) and total bilirubin (TBIL). Additionally, we collected relevant demographic variables (age, sex).

### Annual assessment of liver fibrosis and assessment of HCC risk by FIB-4 index

Liver fibrosis was assessed semiannually by the FIB-4 index^24^, calculated based on the following laboratory values: $\text{ AST }(U/L)\times \text{ age }(\text{ years })/PLT\left(10^{9}/L\right)\times \text{ ALT }(U/L{)}^{1/2}$ . Patients were divided into three groups based on the following FIB-4 index values: < 1.45, 1.45–3.25, and ≥ 3.25, which have been defined as mild (F0-F1), moderate (F2-F3), and advanced (F4) liver fibrosis, respectively, in patients with chronic HBV infection^25^.

HCC incidence was first assessed based on the FIB-4 index at enrollment and then based on updated FIB-4 index values calculated semiannually thereafter. These FIB-4 recalculations and HCC incidence reassessments were repeated within four years from enrollment.

### Statistical analysis

Nonnormal distribution continuous data are presented as median (interquartile range; IQR), and categorical variables as number (percentage; %). Statistical differences between groups were estimated by Mann-Whitney’s test (for nonnormal distribution continuous data) and Chi-squared (for categorical variables) test. Association between the FIB-4 index distribution and the number of years since enrollment was analyzed using the Cochran-Armitage’s test. HCC incidence was calculated using the Kaplan-Meier curve. Comparisons were estimated by the log-rank method. All statistical analyses used R version 4.1.2 and JMP Clinical version 8.1. *P* < 0.05 was considered statistically significant, and all *P*-values were tested by bilateral testing.

## RESULTS

### Baseline patient characteristics

Tables 1 and 2 summarizes the baseline characteristics of this study population. A total of 501 patients participated in the study, of which 27 developed HCC during follow-up. HCC incidences with FIB-4 index < 1.45, 1.45–3.25, and ≥ 3.25 were 1.18%, 1.32%, and 9.09%, respectively (Table 1). CHB patients included 362 men (72.3%) and 139 women (27.7%), with a median age of 42 years (33–51 years). Median PLT level at enrollment was 149 × 10^9^/L, and the median ALT, AST, GGT, ALB, ALP, and TBIL serum were 92 U/L, 69 U/L, 64 U/L, 41.5 g/L, 97 U/L, and 17.9 μmol/L.

**Table 1:** HCC incidence by group

FIB-4 index	Number of HCC	Number of total	HCC incidence
< 1.45	1	85	1.18%
1.45-3.25	2	152	1.32%
≥ 3.25	24	264	9.09%

**Table 2:** Baseline patient characteristics

Characteristic	Nº (%) or Median (IQR)
Age, years	42 (33-51)
Sex (male)	362 (72.3)
Baseline ALT (U/L)	92 (30–352)
Baseline AST (U/L)	69 (33–227)
Baseline PLT (10^9^/L)	149 (113–171)
Baseline GGT (U/L)	64 (29–138)
Baseline ALB (g/L)	41.5 (36.8–45)
Baseline ALP (U/L)	97 (81–133)
Baseline TBIL (μmol/L)	17.9 (11.8–47.6)

#### Annual changes in liver fibrosis assessed by the FIB-4 index

**Figure 1:**
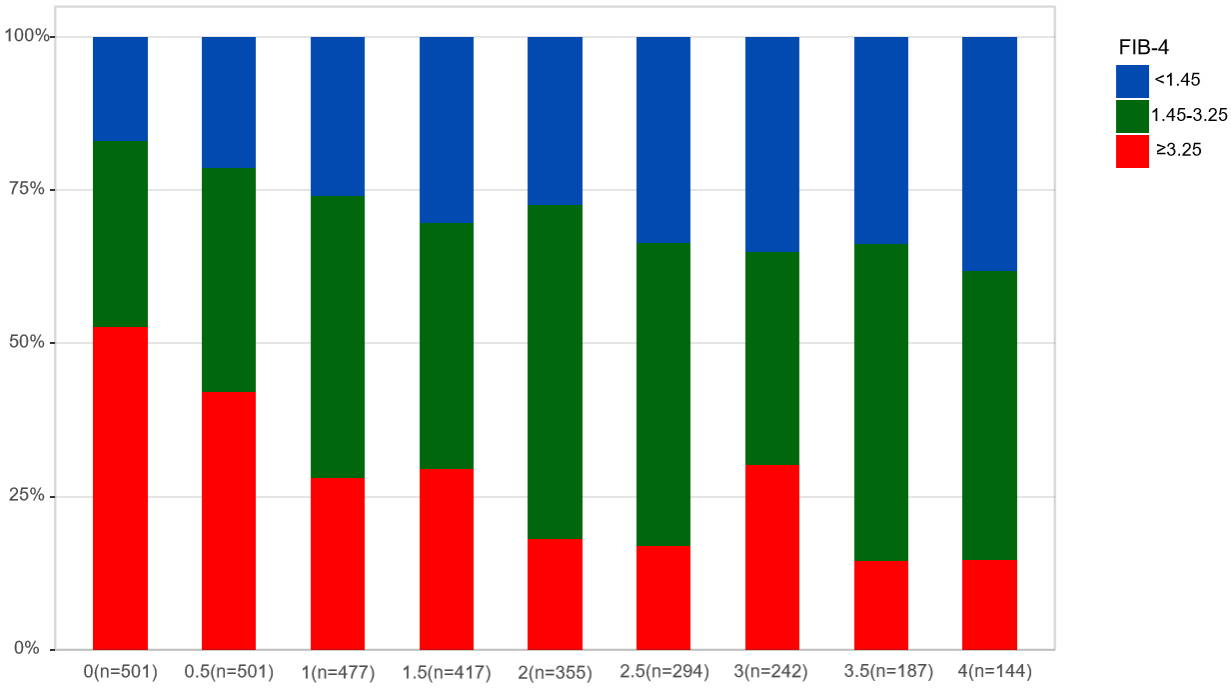
Changes in the distribution of patients with mild, moderate, or advanced fibrosis based on the Fibrosis-4 Index for Liver Fibrosis.

**Figure 2:**
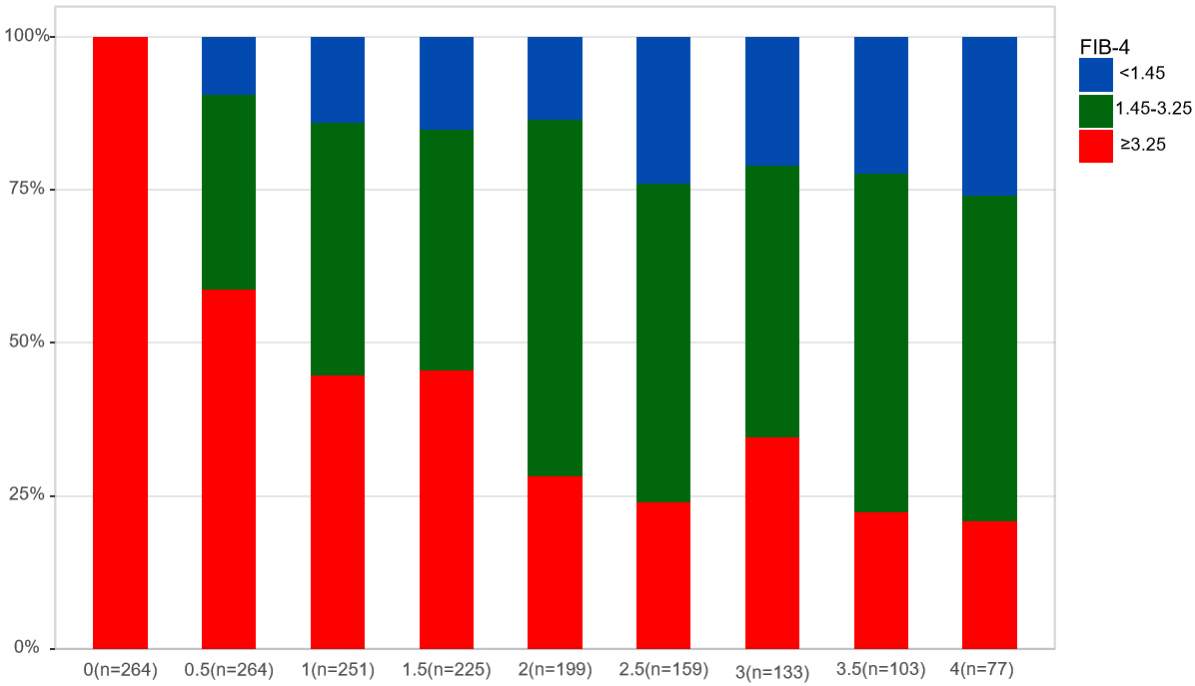
Changes in the distribution of patients with mild, moderate, or advanced fibrosis based on the FIB-4 index among patients with advanced liver fibrosis (FIB-4 index ≥3.25).

Patients were monitored for a median of 2.5 years (1.5-4 years) after enrollment, and 27 patients were excluded from the cohort at the time of HCC diagnosis. Figure 1 shows the annual change in the FIB-4 index distribution. The percentage of patients with a FIB-4 index ≥ 3.25 decreased significantly every six months (*P* < 0.001), going from 52.69% assessed at enrollment to 14.58% at four years. Conversely, the percentage of patients with a FIB-4 index < 1.45 increased significantly (*P* < 0.001), going from 16.97% identified at enrollment to 38.19% at four years. When focusing on patients with advanced liver fibrosis (FIB-4 index ≥ 3.25) at enrollment, the condition gradually resolved within four years in most patients (Figure 2, *P* < 0.001).

#### Longitudinal markers associated with liver fibrosis development

**Figure 3:**
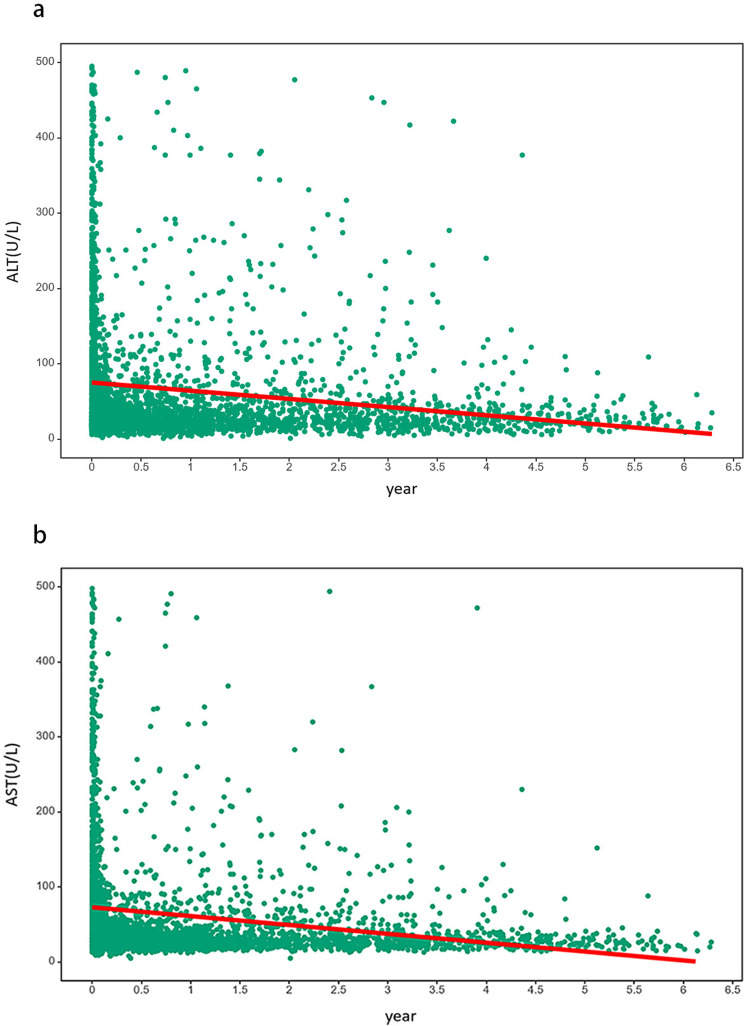
Individual and average development trend graphs of longitudinal variables: a) ALT; b) AST.

Figure 3A and Figure 3B illustrate the average trend for ALT and AST, respectively. The red line represents the variable average trend, and the green dots represent the differences between individuals. Both ALT and AST levels showed a downward trend during the follow-up period.

#### Incidence of hepatocellular carcinoma based on semiannually updated FIB-4 index

**Figure 4:**
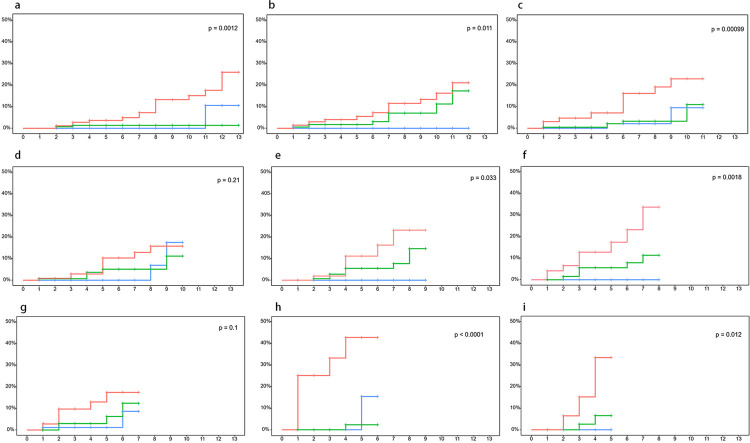
Incidence of hepatocellular carcinoma based on semiannually updated FIB-4 index: a) 0 year; b) 0.5 year; c) 1 years; d) 1.5 years; e) 2 years; f) 2.5 years; g) 3 years; h) 3.5 years; i) 4 years; red lines = FIB-4 index ≥ 3.25; green lines = FIB-4 index 1.45–3.25; blue lines = FIB-4 index < 1.45.

Figure 4 shows the HCC incidence estimated based on the FIB-4 index after enrollment: FIB-4 index update at enrollment (Figure 4A) and at baseline followed by the new assessment date (Figure 4B-4I). The HCC incidence assessed at enrollment and at 0.5, 1, 2, 2.5, 3.5, and 4 years differed significantly between the three groups (*P* < 0.05). Patients with FIB-4 index ≥ 3.25 had the highest HCC incidence, followed by patients with FIB-4 index of 1.45–3.25, and patients with FIB-4 index < 1.45. Patients with FIB-4 index ≥ 3.25 had a higher risk of developing HCC at all time points.

## DISCUSSION

Despite recent evidence that potent NAs therapy may induce regression of liver fibrosis, a small percentage of patients still develop this condition, providing a greater risk for HCC development^15,16,26,27^. It therefore becomes particularly important to assess the fibrosis stage in patients with CHB to establish an effective HCC monitoring system. Although the serological indicators-based FIB-4 index offers only a rough estimate of liver fibrosis and lesser accuracy than liver biopsy imaging diagnosis, it can be repeatedly monitored and is cost-effective. Thus, the FIB-4 can be used to dynamically assess the risk of CHB patients treated with NAs developing HCC.

Results showed a downward trend in both ALT and AST levels during follow-up, which were in line with a previous study^28^. We also assessed changes in liver fibrosis in patients with CHB treated with NAs based on variations in the FIB-4 index and HCC incidence. Our findings showed that after NAs treatment, the percentage of patients with mild liver fibrosis increased, whereas that of patients with advanced liver fibrosis gradually decreased. Although the FIB-4 index calculation includes patient’s age, the number of patients with low FIB-4 index after NAs treatment continued to increase as the patient’s age increased year by year. This trend is consistent with previous studies using histological assessment of liver fibrosis following NAs treatment^18,25,29,30^. Research shows that the FIB-4 index is independently associated with HCC incidence after NAs treatment^31^.

When patients presented a higher FIB-4 index (advanced liver fibrosis), the HCC incidence was also higher. Although the number of patients in the FIB-4 ≥ 3.25 and < 1.45 groups changed at enrollment and at semiannual reassessments, HCC incidence was consistently higher in the former than in the latter. The percentage of patients with FIB-4 index ≥ 3.25 decreased after NAs treatment; however, their risk of developing HCC remained high even after ≥ 4 years. This finding corroborates those of a study in which advanced liver fibrosis was associated with an increased risk of HCC development^32^.

When focusing on patients with advanced liver fibrosis during enrollment, we found that most patients experienced fibrosis regression after undergoing NAs treatment for four years. Additionally, HCC incidence rate remained low in patients with FIB-4 < 1.45 after receiving NAs treatment, suggesting that the risk of developing HCC is relatively low in this population. In patients with a FIB-4 index lower than this value, laboratory testing and ultrasonography can be used for HCC monitoring at slightly longer intervals. Similarly, if the FIB-4 index is ≥3.25, a more intensive HCC monitoring must be implemented in this population. Since the percentage of patients with FIB-4 < 1.45 after receiving NAs treatment increases over time, we could appropriately reduce HCC monitoring and extend the monitoring interval, thus effectively reducing the medical burden of liver fibrosis patients.

### Limitations

This study has several limitations. Firstly, it analyzes patients who underwent NAs treatment but lacks data on patients who did not receive it, thus hindering calculation of the long-term HCC incidence rate in untreated patients. Secondly, the critical FIB-4 index values for diagnosing mild, moderate, and advanced fibrosis are based on studies conducted with patients with CHC, whereas our study was conducted with patients with CHB, thus diagnostic accuracy may be reduced. However, the World Health Organization and the Guidelines for the Prevention and Treatment of CHB have since recommended this index for evaluating liver fibrosis in CHB patients.

## CONCLUSION

Hepatic fibrosis assessment in patients with CHB based on the FIB-4 index showed that the percentage of patients with mild hepatic fibrosis increased, whereas the percentage of patients with advanced hepatic fibrosis decreased after undergoing NAs treatment. Since the risk of developing HCC remains in patients with liver fibrosis, even after the condition slows down, regular monitoring should be conducted for early implementation of intervention measures to reduce the risk of HCC.
